# Comparison of force loss during sliding of low friction and conventional TMA orthodontic archwires: An in vitro study

**DOI:** 10.1007/s00056-020-00266-y

**Published:** 2020-12-02

**Authors:** Nouf Alsabti, Christoph Bourauel, Nabeel Talic

**Affiliations:** 1 Department of Pediatric Dentistry and Orthodontics, College of Dentistry, King Saud University, Riyadh, Saudi Arabia; 2 Oral Technology, Center of Dento-Maxillo-Facial Medicine, University of Bonn, Bonn, Germany

**Keywords:** Tooth movement, Orthodontic brackets, Stainless steel, Titanium molybdenum alloy, Orthodontic friction, Zahnbewegung, Kieferorthopädische Brackets, Edelstahl, Titan-Molybdän-Legierung, Kieferorthopädische Friktion

## Abstract

**Objective:**

The goal was to measure and compare the amount of force loss during tooth movement guided by archwires, including a newly introduced low-friction titanium molybdenum alloy (TMA), conventional TMA, and stainless steel archwires.

**Methods:**

The force loss was measured using a specialized biomechanical set-up, the orthodontic measurement and simulation system (OMSS). A total of 30 specimen were used (10 low-friction TMA (TMA-Low), 10 conventional TMA (TMA-C), and 10 stainless steel (SS) archwires, each having a dimension of 0.016 × 0.022 inches). The conventional and low friction TMA archwires served as test groups, while the SS archwires served as the control group.

**Results:**

The mean values of force loss between the three types of wires (TMA‑C, TMA-Low, and SS) were significantly different (*p* < 0.0001). The highest mean force loss during sliding movement was found in the conventional TMA group (72.1%), followed by low friction TMA (48.8%) and stainless steel wires (33.7%) in a descending order.

**Conclusion:**

The friction property of the low friction TMA archwire was superior to the conventional TMA archwire but was still inferior to the stainless steel archwire.

## Abstract (de)

**Zielsetzung:**

Ziel war es, den Kraftverlust während der bogengeführten Zahnbewegung zu messen und zu vergleichen. Verwendet wurden Bögen aus einer kürzlich eingeführten reibungsarmen Titan-Molybdän-Legierung (TMA) mit konventioneller TMA und Edelstahl.

**Methoden:**

Der Kraftverlust wurde mit einem speziellen biomechanischen Aufbau, dem kieferorthopädischen Mess- und Simulationssystem (OMSS), gemessen. Insgesamt wurden 30 Proben verwendet (10 reibungsarme TMA (TMA-Low), 10 konventionelle TMA (TMA-C) und 10 aus rostfreiem Stahl (SS), jeweils mit einer Dimension von 0,016 × 0,022 Inch). Die konventionellen und reibungsarmen TMA-Bögen dienten als Testgruppen, die SS-Bögen als Kontrollgruppe.

**Ergebnisse:**

Die Mittelwerte des Kraftverlusts zwischen den 3 Drahttypen (TMA‑C, TMA-Low und SS) waren signifikant unterschiedlich (*p* < 0,0001). Der höchste mittlere Kraftverlust während der Gleitbewegung wurde in der konventionellen TMA-Gruppe (72,1%) gefunden, gefolgt von reibungsarmem TMA- (48,8%) und SS-Drähten (33,7%) – in absteigender Reihenfolge.

**Schlussfolgerung:**

Die Reibungseigenschaft des reibungsarmen TMA-Bogens war der des konventionellen TMA-Bogens überlegen, aber immer noch der des Edelstahlbogens unterlegen.

## Introduction

Sliding mechanics for orthodontic space closure is a convenient technique that is widely accepted by many orthodontic clinicians. Thus, knowing the role of friction during treatment is a subject of concern for many orthodontists, as the friction generated between the bracket–archwire interface is integral to the time required for space closure. In orthodontics, friction is considered a clinical challenge especially if sliding mechanics are utilized and it must be controlled efficiently to ensure optimum orthodontic outcomes [1, 11, 24]. Friction is defined as the force that resists movement of one surface against another when two surfaces come into contact [8, 13]. When two surfaces slide, one over the other, an interacting force with two components results: a force perpendicular to the contacting surface, which is the actual force applied (N), and a force tangential to the contacting surface, which is the frictional force (F). The ratio of the tangential force to the applied force is called the coefficient of friction (μ), and it varies according to the nature of the two surfaces and their superficial characteristics. The magnitude of the frictional force is directly proportional to the applied force and dependent on the coefficient of friction such that F = μN [8, 13]. When friction-based mechanics (sliding mechanics) are utilized, a frictional force is generated between the bracket, archwire, and ligature, impeding tooth movement during the retraction phase and transmitting forces to the posterior teeth, thus, negatively affecting the anchorage requirement and resulting in potential loss of anchorage [13]. A percentage of the orthodontic force applied to teeth is lost due to friction when the orthodontic wire slides through the bracket slot and tubes, especially during the space closure stage [21]. It has been estimated that the percentage of the applied force that is lost due to friction is approximately 50%, which means that the total force applied by the clinician has to be twice as large in order to deliver an effective force in the absence of friction [23]. However, the application of heavy forces is nonproductive due to the outcomes of more friction and the risk of anchorage loss [15, 20].

TMA archwires generally have higher frictional properties when compared to other selected alloys which could be attributed to the high reactivity of the wire surface and the production of adhesive and abrasive wear [16]. Stainless steel (SS) archwires are widely used and known for their low friction properties compared to other orthodontic archwire materials [28]. Conventionally, the use of a combination of SS archwires and brackets has been the gold standard for the orthodontists to utilize during sliding mechanics. SS archwires are also considered to be the reference material for researchers assessing the mechanical properties of newly introduced archwires [26]. Given their strength, rigidity, and low-friction smooth-surface characteristics, SS archwires remain the mainstay of orthodontic mechanotherapy [14].

Over the past 100 years, developments in both mechanotherapy and treatment philosophy have resulted in huge improvements in all aspects of patient care. New advances have led to the development and introduction of new orthodontic materials with different properties, adding versatility to orthodontic treatment. Archwire materials comprise a significant part of this change and knowing which wire is appropriate for treatment requires a comprehensive understanding of the archwire biomechanical properties for certain clinical applications. Low-friction TMA wires were introduced in 2014 by the company Ormco to improve the properties of the conventional TMA wires. The company claimed that they used technical innovations to develop new low friction materials by altering the surface treatment and refining the TMA manufacturing process. By changing the processing at the vendor the surface finish was optimized and the frictional properties were improved. These low friction archwires seem to be beneficial to minimize the amount of friction in specific clinical applications. However, the cost for these materials is still considerably higher than for the traditionally used materials and their real cost–benefit ratio remains scientifically questionable.

The aim of this study was to measure and compare the amount of force loss in tooth movement guided by archwires, including a newly introduced low-friction titanium molybdenum alloy (TMA), conventional TMA, and stainless steel archwires.

## Materials and methods

Ethical approval was obtained from King Saud University, College of Dentistry Research Center (PR 0073).

### Sample description

A total of 30 archwires (10 low-friction TMA (TMA-Low), 10 conventional TMA (TMA-C), and 10 stainless steel (SS), from Ormco, Orange, CA, USA; 0.016 × 0.022″ (inch) each) were used. The conventional and low friction TMA archwires served as the test groups, while the stainless steel archwire served as the control group. Stainless steel brackets (0.018″, Roth prescription) were used. The brackets were conventionally ligated to the different archwires using stainless steel ligatures (Table 1).

**Table 1:** Sample description Beschreibung der Proben

Appliance	Product	Dimension (inches)	Manufacturer/Location
*Archwire*			
Stainless steel archwire	Package of Kleen Pack™ system	0.016 × 0.022″ (Preformed)	(Ormco, Glendora, CA, USA)
Conventional titanium molybdenum archwire	TMA® Package of Kleen Pack™ system	0.016 × 0.022″ (Preformed)	(Ormco, Glendora, CA, USA)
Low friction titanium molybdenum archwire	Low Friction TMA® Package of Kleen Pack™ system	0.016 × 0.022″ (Preformed)	(Ormco, Glendora, CA, USA)
*Bracket*			
Stainless steel bracket, (Roth prescription)	Victory series	0.018″	(3M Unitek, Monrovia, CA, USA)
*Ligature*			
Stainless steel ligature	–	0.010″	(Ormco, Glendora, CA, USA)

*TMA* titanium molybdenum alloy

### Experimental setup

**Fig. 1:**
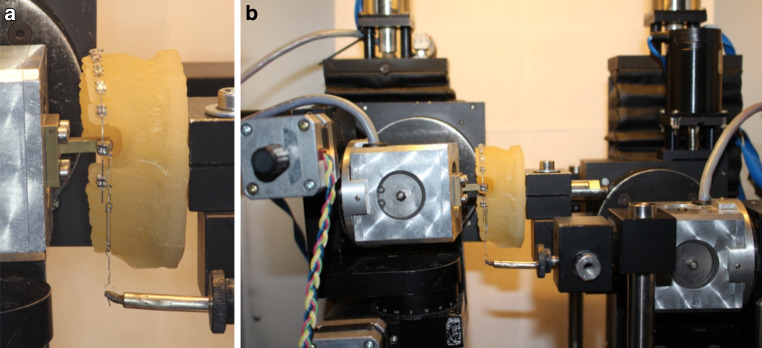
Photograph of the model and canine bracket attached to the OMSS (orthodontic measurement and simulation system) sensors (**b**) with the force applied by a NiTi coil spring (**a**) Aufnahme des Modells und des an den OMSS(kieferorthopädisches Mess- und Simulationssystem)-Sensoren befestigten Eckzahn-Brackets (**b**) mit der durch eine NiTi-Schraubenfeder ausgeübten Kraft (**a**)

A resin replica model of a normally aligned upper arch (Palavit G 4004, Heraeus Kulzer, Hanau, Germany) constructed from a Frasaco model (Franz Sachs, Tettnang, Germany) was used. A normally aligned upper arch model was selected because the space closure and sliding stage typically starts when complete leveling and alignment of teeth is achieved. Stainless steel brackets (0.018″, Roth prescription) were used and bonded to the resin model from the second premolar on one side to the second premolar on the opposite side using cyanoacrylate adhesive. All teeth were bonded except for the extracted canine and first premolar teeth on the left side. For standardization purpose all the bonding procedures were carried out by one investigator. Following the complete bonding procedure, the model was mounted on the Orthodontic Measurement and Simulation System (OMSS; Fig. 1). The OMSS comprises two force–moment sensors mounted on motor-driven positioning tables with three-dimensional mobility, thus, enabling the system to simultaneously measure forces and moments in all three spatial planes acting on the bracket. All the mechanical components of the system are built in a temperature-controlled chamber which is especially important when testing temperature-dependent wires. The components are connected to a personal computer, through which orthodontic tooth movement simulation directions are delivered [9]. The OMSS utilizes a personal computer connected to two microcomputer-based sensors to deliver the commands needed to initiate the experiment and the digital output of the force–moment vectors to the control software.

A bracket holder was fixed to the first sensor where the upper left canine bracket was bonded. The sensor was then adjusted in the position of the upper left canine in the resin model. After that, the wire was ligated to the brackets. All the wires were ligated by the same investigator. Stainless steel ligatures (0.010″) were used for ligation using a needle holder. During ligation of the archwire to the canine bracket, the ligature was initially ligated tightly around the canine bracket wings then loosened by 90° to reduce the pressure generated by the ligature on the wire and permit free movement of the bracket along the archwire. After that, the initial forces and moments generated by the guiding arch on the canine bracket were recorded by the sensor and manually adjusted to be as close to zero as possible.

Over the second sensor, a closed Sentalloy nickel-titanium (NiTi) coil spring (GAC, Central Islip, NY, USA) was attached and hooked over the upper left canine bracket to apply about 1 N of retraction force (approximately 101 g, Fig. 1). The center of resistance of the retracted canine was set by the system software at 8.5 mm apical to the bracket and 4.5 mm lingual to it [18, 27]. The location of the center of resistance in relation to the bracket critically affects the movement of the tooth by producing moments of forces. The software utilized by the OMSS automatically calculates these moments from the measured force system at the bracket and transforms this into the simulated tooth movement that is subsequently executed by the positioning tables of the OMSS.

The OMSS then performed the simulation of the canine sliding movement by dividing it into a maximum of 200 movement increments along an approximately 4 mm retraction path. Each increment consisted of a cycle that starts with simultaneous measurement of the forces and moments delivered by the NiTi coil spring and those acting on the canine bracket. The resultant tooth movement from these forces was then calculated by the system and the canine bracket was retracted along the premolar extraction space. The amount of force loss during each movement increment was calculated as the difference between the force applied by the coil spring and the actual force reaching the canine bracket and used to commence the movement as detailed elsewhere [9]. Wire change and readjustment of the system was performed after each successful simulation run, until all 10 specimens per group were measured.

## Statistical analysis

The statistical analysis was undertaken using SPSS software (IBM SPSS Inc., version 20, Chicago, IL, USA) and the level of significance was set at *P* < 0.05. Assuming an effect size of f = 0.6 [7] with α = 0.05 and β = 0.20 (power 80%), the required sample size was estimated to be 10 specimens in each of the three groups. Descriptive data including the means, standard deviations, minimum and maximum readings were calculated for all groups’ comparison. The differences between the weighted means of force loss percentage were analyzed with one-way analysis of variance (ANOVA) and group differences were further analyzed with Tukey’s post hoc comparisons test.

## Results

**Fig. 2:**
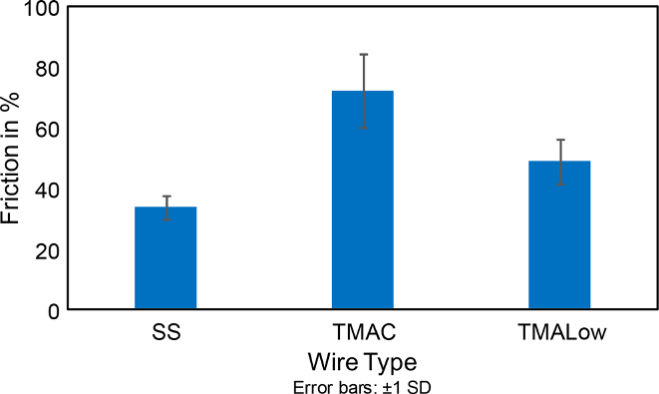
Bar graph of force loss during archwire-guided bracket movement. *SS* stainless steel archwire, *TMAC* conventional titanium molybdenum alloy (TMA) archwire, *TMALow* low-friction TMA archwire, *SD* standard deviation Balkendiagramm des Kraftverlusts während der bogendrahtgeführten Bracketbewegung. *SS *Edelstahlbogen, *TMAC* Bogen aus konventioneller Titan-Molybdän-Legierung (TMA), *TMALow* reibungsarmer TMA-Bogen, *SD* Standardabweichung

Descriptive statistics showed that the highest mean of force loss of 72.1% during sliding movement was found in the TMA‑C group followed by TMA-Low with 48.8% and SS archwires with 33.7% in descending order (Fig. 2, Table 2). The mean values of force loss across the three type of wires (TMA‑C, TMA-Low, and SS) were significantly different (*p* < 0.0001; Table 3). The pairwise comparisons among the three mean values indicated that the mean force loss value of TMA‑C was significantly higher than the mean values of the SS and TMA-Low wires (Table 4).

**Table 2:** Descriptive statistics of force loss with regard to wire type Beschreibende Statistik des Kraftverlustes in Abhängigkeit vom Drahttyp

Wire type	*N*	Mean	Std. deviation	Minimum	Maximum
SS	10	33.7	3.9	27.9	38.5
TMA‑C	10	72.1	12.2	53.5	86.3
TMA-Low	10	48.8	7.4	43.1	69.0
Total	30	51.5	18.0	27.9	86.3

*SS* stainless steel archwire, *TMA‑C* conventional titanium molybdenum alloy (TMA) archwire, *TMA-Low* low-friction TMA archwire, *Std. deviation* standard deviation

**Table 3:** One-way analysis of variance of force loss with regard to wire type Einfaktorielle Varianzanalyse des Kraftverlustes in Abhängigkeit vom Drahttyp

	Sum of squares	Dff	Mean square	F‑value	*p*-value
Between groups	7464.568	2	3732.284	51.273	<0.0001
Within groups	1965.404	27	72.793	–	–
Total	9429.972	29	–	–	–

**Table 4:** Multiple comparisons of mean values of force loss with regard to wire type Mehrfachvergleiche der Mittelwerte der Kraftverluste in Abhängigkeit vom Drahttyp

(I) Type of wire	(J) Type of wire	Mean Difference (I−J) in %	Std. error	*p*-value	95% confidence interval	
					Lower bound	Upper bound
SS	TMA‑C	−38.3^*^	3.8	0.0001	−47.8	−28.9
	TMA-Low	−15.1^*^	3.8	0.001	−24.5	−5.6
TMA‑C	SS	38.3^*^	3.8	0.0001	28. 9	47.8
	TMA-Low	23.3^*^	3.8	0.0001	13.8	32.7
TMA-Low	SS	15.1^*^	3.8	0.001	5.6	24.5
	TMA‑C	−23.3^*^	3.8	0.0001	−32.7	−13.8

*The mean difference is significant at the 0.05 level

*SS* stainless steel archwire, *TMA‑C* conventional titanium molybdenum alloy (TMA) archwire, *TMA-Low* low-friction TMA archwire, *Std. error* standard error

## Discussion

Friction in orthodontic treatment is considered a clinical challenge, especially with sliding mechanics and it must be well understood and controlled since this component cannot be eliminated from the materials. Because previous studies showed that TMA archwires exhibited significantly higher frictional values during sliding mechanics when compared to stainless steel wires [2, 8, 13, 14, 25, 26], the manufacturers introduced TMA archwires with different surface treatments attempting to reduce their frictional characteristics [6].

The results of the present study showed that mean values of force loss of three archwires were significantly different from each other. The mean force loss value for the TMA‑C group was found to be significantly higher than the mean values of the other two wires (SS and TMA-Low). This is in agreement with previous studies that showed high frictional values associated with TMA wires during sliding mechanics [2, 8, 13, 14, 25, 26]. The standard deviation in the obtained TMA‑C data is considered high which is due to the extremely high numbers in the data set. This could be due to a high variability of the archwires surface finish as TMA wires tend to show different patterns of surface irregularities due to the complex manufacturing process [3, 19, 22].

The TMA-Low archwire had an intermediate value of force loss between the other two groups (TMA‑C and SS archwires). The results indicate that the amount of force loss in the TMA-Low wire was significantly lower and higher than in the TMA‑C and SS groups, respectively. Although our findings indicate that the TMA-Low archwire exhibits less force loss during sliding when compared to the TMA‑C archwire, it is still considered inferior to the stainless steel archwire. This is in accordance with a reported study that evaluated the properties of the newly introduced TiMolium alloy which is a modified TMA alloy containing both aluminum and vanadium as stabilizing agents to improve the mechanical properties of the conventional TMA alloy. It was found that this alloy provided better strength, less friction, and smoother surface features than conventional TMA alloys. However, it was still inferior to SS archwires [14]. In addition, colored TMA archwires were introduced by passing a direct electrical current through the titanium alloy in order to have an improved surface finish and to reduce the frictional characteristics of the wire [6]. Furthermore, ion implanted TMA is another modified TMA archwire that was treated by accelerating the vapor flux of ions against the archwire via an electron beam evaporator to improve the surface finish as the ions penetrate the surface [6]. Evaluating the frictional characteristics of these archwires has shown that the ion implanted TMA wires showed minimum frictional forces and most of the colored TMA archwires demonstrated frictional characteristics similar to the conventional TMA archwire with both groups having no advantage over the stainless steel archwire group [6].

Among the three mean values, the SS wire had a lower mean value of force loss when compared with the other two TMA wires (TMA‑C and TMA-Low). In another previous study, it was found that SS wires generated the least amount of friction, followed by cobalt–chromium (CoCr), nickel–titanium (NiTi), and beta-titanium (β-Ti) wires [25]. These findings are consistent with those of multiple studies that have found that SS wires produce the least amount of friction compared with other alloys [13, 17].

In our study, a stainless steel ligature was used to ligate the upper left canine bracket for two reasons. First, many authors agreed that using loosely tied stainless steel ligatures generate less friction when compared to the standard elastomeric ligatures [10, 12]. Second and more importantly, using a stainless steel ligature allowed to control the force of ligation and minimize the frictional forces generated by the ligature on the wire. By loosening the ligature by 90°, it was ensured that the force applied on the canine bracket over the first sensor equals the force generated by the attached NiTi coil over the second sensor. Equal forces on the two sensors ensured that no additional frictional forces from the steel ligature were placed over the first sensor. During upper left canine bracket ligation and NiTi coil attachment, the amount of forces in the Z direction in both sensors representing the generated and applied forces were monitored via the connected personal computer. The simulation of tooth movement was not initiated until the forces appeared equal by adjusting the sensors to eliminate any additional forces resulting from the ligation procedure.

The experimental set-up of any study has a critical effect on the resulting outcomes. Multiple previous studies evaluating frictional values have utilized a testing model designed without representing the complete dental arch [4, 5, 13]. In our study, the OMSS device was used which utilizes a testing model representing the complete dental arch. The set-up included a resin replica where archwire-guided retraction of the canine bracket was performed simulating the clinical tooth retraction during sliding mechanics in which the position of the center of resistance (CR) of the retracted bracket is located away from the bracket’s plane. The OMSS enables any anticipated static and dynamic orthodontic movement to be analyzed at the level of a two-tooth model [9]. As the measurements are dynamic, the simulated tooth resembles the clinical reaction during sliding with tipping and rotation until the wire gets deformed and produces counteracting moments. The experimental set-up used in this study allowed for easy observation of the amount of force loss among the three alloys simulating the actual clinical situation during canine retraction. As a result, the TMA‑C arch showed the highest value of force loss followed by the TMA-Low and stainless steel wires.

## Conclusions

The differences of force loss during sliding movement between the three types of wire were significant, i.e., the resistance of sliding of the newly introduced TMA-Low archwire was superior to the TMA-C archwire but inferior to the SS archwire.
